# Nonlinear coupling in graphene-coated nanowires

**DOI:** 10.1038/srep38924

**Published:** 2016-12-12

**Authors:** Yixiao Gao, Ilya V. Shadrivov

**Affiliations:** 1 Key Lab of All Optical Network & Advanced Telecommunication Network of EMC, Institute of Lightwave Technology, Beijing Jiaotong University, Beijing 100044, China; 2 Nonlinear Physics Centre, Research School of Physics and Engineering, The Australian National University, Canberra ACT 2601, Australia

## Abstract

We propose and analyze nonlinear coupler based on a pair of single mode graphene-coated nanowires. Nonlinear wave interactions in such structure are analyzed by the coupled mode equations derived from the unconjugated Lorentz reciprocity theorem. We show that the routing of plasmons in the proposed structure can be controlled by the input power due to the third order nonlinear response of graphene layer. Our findings show that graphene nonlinearity can be exploited in tunable nanoplasmonic circuits based on low-loss, edgeless cylindrical graphene waveguides.

Light control at the nanoscale is one of the important aims of the rapidly developing research field of nanophotonics. Conventional silicon-based photonic circuits cannot localize and manipulate light at the nanoscale due to the diffraction nature of light^1^. Surface plasmon, a hybrid wave which couples light and free electron oscillations at metal-dielectric interfaces, is among the most promising candidates for subwavelength light manipulation^1^. In long wavelength infrared and terahertz frequencies, graphene supports highly confined, low-loss surface plasmon waves with the possibility to achieve active tunability through electrical gating^2^. Graphene nanoribbons have been considered as potential building blocks for future large-scale integration optoelectronics^3^. The fundamental edge mode of graphene nanoribbon is highly localized near the ribbon edge^3^,^4^, and this can cause additional scattering loss due to any defects of the edge structure^5^. To avoid this problem, graphene coated nanowires (GNW) are proposed for efficient surface plasmon waveguiding in plasmonic nanocircuits^6^,^7^. Fabrication of such nanowires is experimentally feasible with current technology for wire diameters ranging from tens of nanometers to several micrometers^8^,^9^,^10^. Although GNW shows superior characteristics as a passive waveguide, the cylindrical graphene structure may pose new challenges to achieve efficient gate-tunability which has been exploited in active planar graphene plasmonic devices.

Graphene has nontrivial nonlinear properties. As an example, strong four wave mixing was experimentally observed in graphene, indicating that it exhibits third order nonlinear response^11^. Wavevector-induced second order nonlinear effects were also studied theoretically and demonstrated experimentally^12^. Nonlinear plasmonic effects predicted in graphene include spatial solitons^13^, nonlinear plasmonic modes^14^, discrete solitons^15^, nonlinear couplers based on graphene sheets^16^, and frequency conversion^17^,^18^. By employing large Kerr-like nonlinear response of graphene, we can realize a series of tunable plasmonic devices through a new degree of freedom, i.e. power of guided waves.

In this paper, we study the nonlinear coupling in a pair of single mode graphene coated nanowires. We first review the properties of the linear modes in a pair of graphene coated nanowires. Then, we derive the coupled mode equations for nonlinear coupling in the structure based on the unconjugated Lorentz reciprocity theorem and solve them numerically. Finally, we demonstrate the power-dependent behaviour of the GNW coupler that allows to route the output by input intensity, and we discuss the influence of structural parameters on coupling.

## Results and Discussion

### Theoretical model

**Figure 1:**
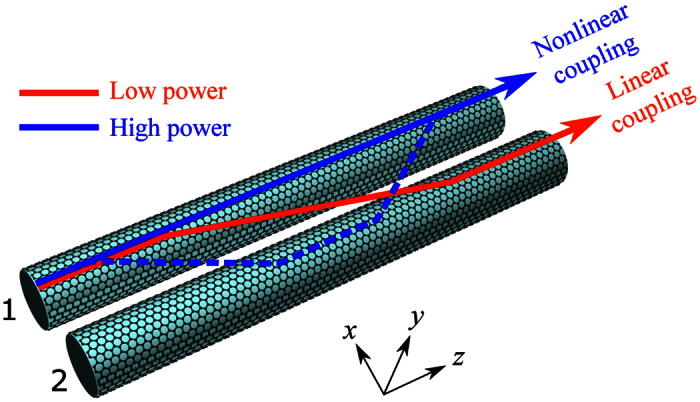
Nonlinear coupler based on a pair of graphene coated nanowires. Red and blue arrows schematically show the direction of power flow for low and high input power, denoting the linear and nonlinear regimes, respectively.

Schematics of the structure is shown in Fig. 1. Two parallel dielectric nanowires of radius *R* are covered by graphene, which is held in place by Van der Waals force^9^. The relative permittivity of the dielectric is *ε* = 2.5, and the spacing between the wires is *d*. For simplicity, it is assumed that the structure is placed in free space.

Graphene is characterized by its surface conductivity as *σ* = *σ*^(1)^ + *σ*^(3)^|**E**_*τ*_|^2^, where **E**_*τ*_ is the electric field tangential to the graphene surface. The linear surface conductivity *σ*^(1)^ is a superposition of an interband and an intraband contributions, which are described by the Kubo formula^19^:


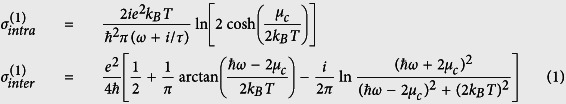


where *e* is electron charge, *μ*_*c*_ is chemical potential, *k*_*B*_ is Boltzmann constant, *τ* is scattering time, and *T* is temperature. We assume that *μ*_*c*_ = 0.5, *τ* = 10 ps, corresponding to low loss high quality graphene^16^,^20^,^21^, and *T* = 300 K.

The third order nonlinear conductivity^22^ *σ*^(3)^ is


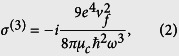


where *v*_*f*_ ≈ *c*/300 is the Fermi velocity.

**Figure 2:**
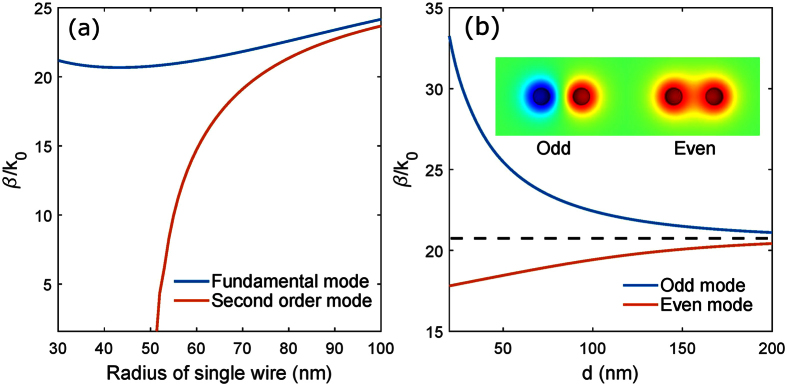
(**a**) Effective mode index of fundamental and second order modes in a single graphene coated nanowire as a function of wire radius. (**b**) Dependence of the wavenumber of the GNW pair modes on the spacing between the wires. Inset shows the mode profile (z-component of electric field) of odd and even modes in GNW pair with R = 50 nm and d = 150 nm. Dashed line shows the wavenumber of fundamental mode of single GNW. Frequency is 30 THz.

A single graphene coated nanowire can support different order modes, which can be classified by azimuthal mode profile^6^. For a GNW of a given radius, the fundamental mode does not have a cut-off frequency, while all the higher order modes do. Alternatively, for a given frequency, existence of the higher order modes requires certain minimum wire radius, while thinner GNWs support only fundamental mode. For the case of directional coupler, we choose both wires in a single mode regime. Figure 2(a) shows the radius-dependent mode index of the first two modes of a single wire at 30 THz. When the nanowire radius reduces to below about 50 nm, the second order mode experiences cut off, allowing us to work in a single mode regime.

When two single-mode GNWs are placed parallel and are close enough, the hybridisation of the modes creates antisymmetric (odd) mode and symmetric (even) mode, as shown in the inset in Fig. 2(b). Figure 2(b) shows the mode index of odd and even modes in GNW pair as a function of distance between wires d. The odd mode has larger mode index than its even counterpart. Any wave propagation in these coupled waveguides in linear regime can be represented as a superposition of the odd and even modes. If both waves are excited, then one will observe beating of the waves, and the beat length is defined by the difference between propagation constants of the two modes: *L*_*B*_ = *π*/Δ*β* with Δ*β* = *β*_*a*_ − *β*_*s*_, *β*_*a*_ and *β*_*s*_ are propagation constants of odd and even modes, respectively.

### Nonlinear coupling

The linear modes in GNW pair can be described as {**e**_*m*_, **h**_*m*_} exp(−*α*_*m*_*z*) exp(*iβ*_*m*_*z*), where *β*_*m*_ is the wavenumber, *α*_*m*_ is the attenuation of the *m*-th mode, {**e**_*m*_, **h**_*m*_} are transverse profiles of electric and magnetic fields, and *m* is either “a” or “s”, denoting the antisymmetric or symmetric mode.

To describe the nonlinear interactions, we aim to derive coupled mode equations^23^,^24^. Considering relatively high loss in plasmonic waveguide, as compared to dielectric waveguides, we employ unconjugated Lorentz reciprocity theorem to derive the coupled mode equations for the nonlinear coupler. For *z*-invariant graphene plasmonic waveguide, the reciprocity theorem^23^ gives


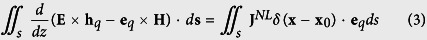


where **x**_0_ denotes the position of graphene layer on the cross section of waveguide, **J**^*NL*^ is the nonlinear surface current in graphene, {**E**, **H**} are the electromagnetic field in the presence of nonlinear current **J**^*NL*^, {**e**_*q*_, **h**_*q*_} is the *q*-th eigen mode of the waveguide, and S is the entire cross-section of the waveguide where the fields are present.

Assuming that the presence of **J**^*NL*^ does not modify the mode profile but only its amplitude, we can write electromagnetic field {**E**, **H**} in such waveguide as a superposition of linear modes. Considering the fact that GNW pair in this paper only supports two modes, we have


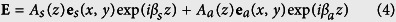


in which *A*_*s*_ and *A*_*a*_ are the amplitude of even and odd modes with a dependence on *z*. Expressions for the magnetic field **H** can be written in a similar way.

Since the even and odd modes originate from the hybridization of TM modes in a single GNW^25^, the *z* component of electric field is much larger than other tangential field components. Then, the induced nonlinear surface current has only z-component and it is expressed as


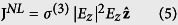


Substituting (4) into (5), we have


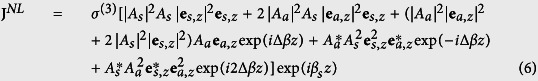


We first consider the amplitude evolution of even mode *A*_*s*_(*z*). Substituting (4) and (6) into (3) and letting 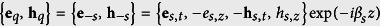, which corresponds to the even mode propagating along -*z*-direction, we have amplitude equation for *A*_*s*_ as


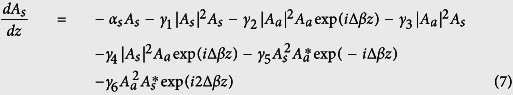


where the nonlinear coupling coefficients are


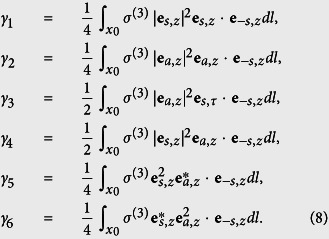


Due to the field symmetry of even and odd modes, *γ*_2_, *γ*_4_ and *γ*_5_ vanish after integration. The amplitude equation for the odd mode *A*_*a*_ could be derived in a similar way. As a result, the coupled mode equations for the nonlinear coupling can be derived as


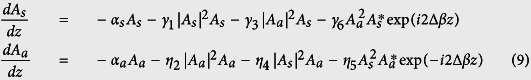


where *η*_*i*_ can be calculated from (8) by replacing **e**_−*s*,*z*_ by **e**_−*a*,*z*_.

To show a particular example, we consider a pair of graphene coated nanowires with *R* = 50 nm, *d* = 150 nm and the working frequency is 30 THz. We calculate eigen mode profiles and their mode indices numerically using commercial simulation package COMSOL. We found that the mode indices of odd and even modes are 21.50 and 20.08, respectively, and this leads to the beat length *L*_*B*_ of 3.52 *μ*m.

**Figure 3:**
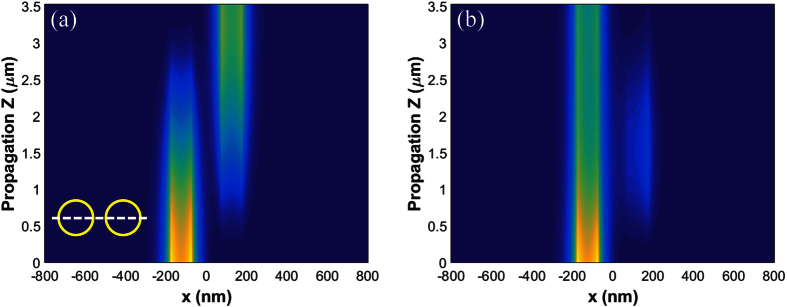
Energy density distribution in (**a**) linear regime, *P*_0_ = 0.01 mW. (**b**) Nonlinear regime, *P*_0_ = 2.9 mW. The wave is launched in the left GNW. Bottom left corner of panel (a) shows cross-section of the pair of waveguides with dashed line indicating the plane on which we plot the spatial distribution of energy density.

Figure 3 illustrates the spatial distribution of energy density on a plane of the GNW axes. We assume that the input wave of the power *P*_0_ is launched into the left nanowire. The input power in Fig. 3(a) is *P*_0_ = 0.01 mW, which is low enough so that all nonlinear terms in equations are negligible, i.e. at least 10 times smaller than linear terms. As the wave propagates, the energy couples from the left nanowire to the right one, with complete energy transfer after propagating a distance of *L*_*B*_. We note that the total energy decreases with propagation due to intrinsic loss in graphene. When the input power is large enough, the coupling changes due to graphene nonlinear response. Figure 3(b) shows the energy density distribution for *P*_0_ = 2.9 mW. After propagating a distance of *L*_*B*_, most of the energy still flows in the left nanowire with little coupling to the right waveguide. At the distance about *L*_*B*_/2, a small portion of energy is coupled into the right wire, and then coupled back to left wire while propagating toward *L*_*B*_.

**Figure 4:**
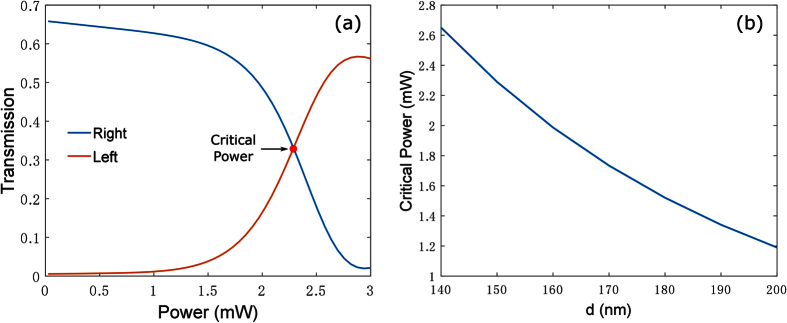
(**a**) Energy flow in the right and left waveguides after propagating the distance *L*_*B*_ as functions of the input power. As in all previous simulations, the input wave is coupled to the left GNW. (**b**) Critical power as a function of spacing d.

To further demonstrate the power dependence of the coupler performance, Fig. 4(a) shows transmitted energy in the left and right waveguides for different input powers coupled to the left wire. With the input power increasing, nonlinear self action switches the output power from the right nanowire into the left nanowire. At the critical power *P*_0,*c*_ = 2.29 mW, equal output power from both wires can be achieved. When *P*_0_ further increased to 2.9 mW, the power is predominantly localized in the left nanowire. It is worth noting that in our simulations, when the input power is 3 mW, the maximum electric field in graphene is 3 × 10^7^ V/m, which is well below the breakdown threshold of graphene^26^.

The spacing d has an important impact on the switching power. Figure 4(b) shows the change of critical power with spacing d. With d increasing, critical power monotonously decreases. This could be explained as follows: larger d results in a larger *L*_*B*_ and vice versa, which can be inferred from Fig. 2(b). As the length of the whole process considered in this paper is *L*_*B*_, a longer coupler length means longer nonlinear interaction region, leading to larger nonlinear phase shift in the coupler and as a result smaller input power is required to achieve the nonlinear switching. At the same time, the length of the coupler cannot be too large, because of graphene absorption and finite plasmon propagation length. Thus we choose an intermediate spacing *d* = 150 nm in this work, which gives *L*_*B*_ that is shorter than the graphene plasmon decay length, and long enough so that the nonlinear effects allow to observe the switching in the coupler.

## Conclusion

In conclusion, we have studied the nonlinear plasmon coupling in a pair of single mode graphene coated nanowires. Based on unconjugated Lorentz reciprocity theorem, we derived the coupled mode equations describing nonlinear wave propagation in such a structure. We demonstrated that the nonlinear switching of plasmons between the waveguides can be achieved by changing the input power, and due to the large third order nonlinearity and tight field confinement, relatively low power is sufficient for achieving efficient switching. Our results shows nonlinearity of graphene could be exploited as a new degree of freedom for designing active plasmonic devices based on cylindrical graphene structure.

## Methods

### Lorentz reciprocity theorem for graphene waveguide

Electromagnetic wave excited by surface current **J** and guided by a GNW pair satisfies Maxwell’s equation


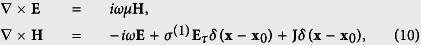


where **x**_0_ is the position vector of graphene layer on the waveguide cross section. The electric and magnetic fields {**e**_*q*_, **h**_*q*_} of the *q*-th eigen mode of GNW pair satisfies the source free Maxwell’s equations:


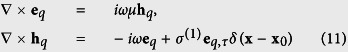


We multiply Eq. (10) by {**h**_*q*_, **e**_*q*_}, respectively. Then, we multiply Eq. (11) by {**H**, **E**}, and combining them we arrive at the reciprocity theorem in the form


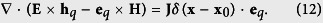


By integrating both parts of this equation over a suitable volume and applying the divergence theorem, taking into account the bounded nature of guided wave, we derive Eq. (3).

### Numerical calculation of modes in GNW pair

Finite element method (FEM) calculations for the linear modes in GNW pair are performed with the help of COMSOL Multiphysics modal analysis with perfectly matched layer (PML) enclosing the structure. Graphene is modelled as an electric field-induced surface current **J** = *σ*^(1)^**E**_*τ*_ on the surface of nanowire.

## Additional Information

**How to cite this article**: Gao, Y. and Shadrivov, I. V. Nonlinear coupling in graphene-coated nanowires. *Sci. Rep.* **6**, 38924; doi: 10.1038/srep38924 (2016).

**Publisher's note:** Springer Nature remains neutral with regard to jurisdictional claims in published maps and institutional affiliations.
